# Cognitive deficits in bipolar disorder type 1: a case-control study

**DOI:** 10.1192/j.eurpsy.2026.10702

**Published:** 2026-07-31

**Authors:** N. Smaoui, H. Sghaier, L. Triki, S. Omri, I. Gassara, M. Maalej, N. Charfi, M. Maalej, R. Feki, L. Zouari

**Affiliations:** 1 Psychiatry “C”, Hedi Chaker University Hospital; 2 Department of Functional Explorations, Habib Bourguiba University Hospital, Sfax, Tunisia

## Abstract

**Introduction:**

Bipolar disorder type 1 (BD-1) is often associated with cognitive deficits, even during remission. These impairments affect memory, attention, and executive functions, impacting daily life. Assessing cognition with the Screen for Cognitive Impairment in Psychiatry (SCIP) helps identify specific deficits and guide interventions.

**Objectives:**

To compare the cognitive performance of patients with bipolar disorder type 1 (BD-1) with that of healthy controls, using the Screen for Cognitive Impairment in Psychiatry (SCIP) scale, in order to identify any specific deficits

**Methods:**

A cross-sectional case-control study was conducted at the Hedi Chaker University Hospital in Sfax, Tunisia, involving 15 patients with TB-1 and 15 controls. Cognitive assessment was performed using the SCIP, analyzing the total score and five subscores: immediate verbal memory (VLT-I), working memory (WMT), verbal fluency (VFT), delayed verbal memory (VLT-D), and processing speed (PST).

**Results:**

The total SCIP score was lower in cases (41.47 ±12.2) than in controls (47.27 ±10.6), without statistical significance (p=0.176). The subscores showed the following differences:

- VLT-I: 14.2 ±4.68 (cases) vs. 15.0 ±4.14 (controls; p=0.624).

- WMT: 15.73 ±3.96 (cases) vs. 18.13 ±3.27 (controls; p=0.081).

- VFT: 4.60 ±2.20 (cases) vs. 5.40 ±2.23 (controls; p=0.331).

- VLT-D: 2.87 ±2.20 (cases) vs. 4.33 ±2.23 (controls; p=0.080).

- PST: 5.53 ±2.39 (cases) vs. 4.40 ±1.92 (controls; p=0.163).

**Conclusions:**

Patients with TB-1 had lower overall cognitive scores than controls, with marked differences in working memory (WMT) and delayed verbal memory (VLT-D). These observations reinforce the hypothesis of cognitive deficits associated with TB, particularly in the areas of memory and executive function.

**Disclosure of Interest:**

None Declared

